# Impact of adolescents’ binge drinking on blood chemistry

**DOI:** 10.1007/s00431-024-05862-3

**Published:** 2024-12-12

**Authors:** Enrico Pistritto, Federica M. F. Schera, Emilia Vassilopoulou, Antonio Corsello, Ilaria Alberti, Sebastiano A. G. Lava, Céline Betti, Mario G. Bianchetti, Carlo Agostoni, Pietro Camozzi, Gregorio P. Milani

**Affiliations:** 1 Pediatric Unit, Fondazione IRCCS Ca’ Granda Ospedale Maggiore Policlinico, Via Della Commenda 9, 20122 Milan, Italy; 2 Department of Clinical Sciences and Community Health, Università Degli Studi Di Milano, Milan, Italy; 3 Family Medicine Institute, Faculty of Biomedical Sciences, Università Della Svizzera Italiana, Lugano, Switzerland; 4 Pediatric Cardiology Unit, Department of Pediatrics, Centre Hospitalier Universitaire Vaudois, and University of Lausanne, Lausanne, Switzerland; 5 Clinical Pharmacology Service, Centre Hospitalier Universitaire Vaudois and University of Lausanne, Lausanne, Switzerland; 6 Institute of Pediatrics of Southern Switzerland, Ente Ospedaliero Cantonale, Bellinzona, Switzerland; 7 Pediatric Emergency Department, University Children’s Hospital Zurich, Zurich, Switzerland; 8 Department of Anesthesia, Hôpital du Valais, Sion, Switzerland

**Keywords:** Acid–base balance, Adolescence, Binge drinking, Glucose, Potassium, Sodium

## Abstract

Adolescent binge drinking is increasingly common. This study investigates the anomalies in glucose, sodium, calcium, potassium, and acid–base homeostasis induced by binge drinking in adolescents. The records of teenagers who sought medical attention for binge drinking (ethanol level ≥ 0.80 g/L) at the Pediatric Emergency Department, Ca’ Granda Ospedale Maggiore Policlinico, Milan (Italy), spanning the years 2013 to 2023 were retrospectively analyzed. For this analysis, cases were selected if documented blood chemistry encompassed sodium, potassium, total calcium, glucose, acid–base balance, and lactic acid (only for those with metabolic acidosis). Included were 173 adolescents (female-to-male ratio 0.94), 13.2 to 18.4, median 16.4 years of age. Hypoglycemia (≤ 3.3 mmol/L; *N* = 1, 0.6%), hyponatremia (≤ 134 mmol/L; *N* = 7, 4.0%), hypernatremia (≥ 146 mmol/L; *N* = 3, 1.7%), hypocalcemia (≤ 2.19 mmol/L; *N* = 0) hypercalcemia (≥ 2.61 mmol/L; *N* = 0), and hyperkalemia (≥ 5.1 mmol/L; *N* = 0) were infrequent. Acute respiratory acidosis (pCO_2_ ≥ 46 mm Hg; pH < 7.40; *N* = 101, 58%) was the most common acid–base imbalance, followed by respiratory alkalosis (pCO_2_ ≤ 34 mm Hg; pH > 7.40; *N* = 10, 5.6%), and metabolic acidosis (HCO_3_^-^ ≤ 19 mmol/L, pH < 7.40; *N* = 9, 5.2%). The lactic acid level was increased (≥ 2.1 mmol/L) in all cases with metabolic acidosis. Metabolic alkalosis (HCO_3_^-^ ≥ 28 mmol/L, pH > 7.40) never occurred. Hypokalemia (≤ 3.4 mmol/L; *N* = 56, 32%) was prevalent, particularly in adolescents with normal acid–base equilibrium or metabolic acidosis, rather than respiratory acidosis or alkalosis.

*Conclusion*: Adolescents who engage in binge drinking often experience a disrupted acid–base balance and hypokalemia, while glucose, sodium and calcium levels are rarely affected.

**Table:** 

**What is known?** • *Binge drinking is becoming increasingly common among adolescents.* • *Conflicting data regarding the type and prevalence of biochemical disorders induced by binge drinking are available in this age group.*
**What is new?** • *Acute respiratory acidosis is prevalent in adolescents with binge drinking, whereas respiratory alkalosis, metabolic acidosis, and hypoglycemia are uncommon.* • *Hypokalemia develops frequently.*

**What is known?**

• *Binge drinking is becoming increasingly common among adolescents.*

• *Conflicting data regarding the type and prevalence of biochemical disorders induced by binge drinking are available in this age group.*

**What is new?**

• *Acute respiratory acidosis is prevalent in adolescents with binge drinking, whereas respiratory alkalosis, metabolic acidosis, and hypoglycemia are uncommon.*

• *Hypokalemia develops frequently.*

## Introduction

The ingestion of ethanol in large amounts can lead to features such as changes in consciousness, seizures, hypothermia as well as disruptions in blood glucose, electrolyte, and acid–base balance [1–4]. In infants and children, acute ethanol consumption is typically inadvertent and unsupervised [1, 3]. This can happen through various sources, including drinking from a glass containing alcohol, exposure to household items like perfumes and hand sanitizers, or ingesting medications that contain alcohol [1, 3]. In teenagers, acute ethanol intoxication mainly results from binge drinking [2, 4].

Binge drinking can occasionally cause a disturbed glucose, electrolyte, and acid–base homeostasis [5–9]. However, the literature provides conflicting results regarding type and prevalence of biochemical disorders induced by binge drinking in adolescents [5–9]. This is partly due to the lack of adequate distinctions between figures obtained in infancy, childhood, adolescence or adulthood, and data collected in individuals who consume ethanol either acutely or chronically [5–9]. Additionally, in various studies, the number of cases included has been limited.

Binge drinking is more and more common among adolescents [2, 4]. The present study delineates clinical information and anomalies related to glucose, sodium, potassium, and pH homeostasis in slightly over 170 adolescents with acute ethanol intoxication.

## Patients and methods

### Study participants and design

The medical records of previously healthy adolescents aged 10 to 18 years, who sought medical attention at the Pediatric Emergency Department of the Fondazione IRCCS Ca’ Granda Ospedale Maggiore Policlinico, Milan, Italy, spanning the years 2013 to 2023 and carrying a diagnosis of binge drinking, were retrospectively assessed. Binge drinking was defined as the consumption of an excessive ethanol quantity within a two-hour interval, leading to the attainment of a blood ethanol concentration level of ≥ 0.80 g/L [2]. Individuals with pre-existing conditions affecting the heart, endocrine system, metabolism, kidneys, or respiratory system were excluded. Additionally, participants receiving medications that could impact electrolyte levels or disrupt acid–base balance were also not considered for inclusion.

For this analysis, we included only cases with documented information on demographics, Glasgow Coma Scale, binge drinking history, concurrent use of illicit substances such as cannabinoid or cocaine, seizures, core temperature, O_2_-saturation, heart rate, blood pressure, and levels of ethanol, albumin, hemoglobin, sodium, chloride, potassium, total calcium, acid–base balance, lactic acid (in metabolic acidosis only), glucose, urea, and creatinine in venous blood, and respiratory support other than O_2_-therapy.

### Laboratory procedures

A Cobas 8000 c702 analyzer was used for the determination of ethanol (enzymatic assay), albumin (bromcresol purple method), total calcium (cresolphtalein complexone method), urea (urease method) and creatinine (Jaffe method) in plasma [10]. A GEM® Premier TM 4000 autoanalyzer was used for the determination of hemoglobin (optical absorbance), ionized sodium, ionized chloride, ionized potassium, pH and carbon dioxide pressure (direct potentiometry), and lactic acid and glucose (amperometry) in whole blood [10]. No systematic urine testing for cannabinoids or cocaine use was carried out.

The concentration of bicarbonate was calculated using the Henderson-Hasselbalch equation [10, 11]. The anion gap was determined by adding the concentrations of sodium and potassium, and then subtracting the sum of the chloride and bicarbonate concentrations from this total [10]. Total osmolarity (in mosm/L) was calculated from sodium (Na^+^), glucose, urea, and ethanol (in mmol/L) using the equation: Na^+^ × 2 + glucose + urea + ethanol [12]. The effective blood osmolarity, which indicates the level of solutes that do not readily cross the cell membrane, was determined from sodium and glucose using the equation: Na^+^ × 2 + glucose [12].

### Reference values—definitions

Altered consciousness was diagnosed with a Glasgow Coma Scale ≤ 12 [13], hypoxemia with O_2_-saturation ≤ 92%, and hypothermia with central body temperature ≤ 35.0 °C.

Normal acid–base balance was diagnosed with pCO_2_ levels of 35–45 mm Hg (4.67–6.00 kPa) and bicarbonate of 20–27 mmol/L [10]. Respiratory acidosis [10] was diagnosed with hypercapnia (≥ 46 mm Hg, respectively ≥ 6.13 kPa) and pH < 7.40, with or without compensatory hyperbicarbonatemia (≥ 28 mmol/L). Respiratory alkalosis [10] was diagnosed with hypocapnia (≤ 34 mm Hg, respectively ≤ 4.53) and pH > 7.40, with or without hypobicarbonatemia (≤ 19 mmol/L). Cases featuring hypobicarbonatemia and pH < 7.40, regardless of hypocapnia, were diagnosed with metabolic acidosis [10]. If L-lactic acid concentration was ≥ 2.1 mmol/L, they were further categorized as lactic acidosis [14]. Metabolic alkalosis [10] was diagnosed with hyperbicarbonatemia (≥ 28 mmol/L) and pH > 7.40, with or without hypercapnia. Hypernatremia was identified with ionized sodium ≥ 146 mmol/L, hyponatremia with ≤ 134 mmol/L [10]; hyperchloremia with chloride ≥ 110 mmol/L, hypochloremia with ≤ 96 mmol/L [10]; hyperkalemia with potassium ≥ 5.1 mmol/L, hypokalemia with ≤ 3.4 mmol/L [10]; hypercalcemia with calcium ≥ 2.61 mmol/L, hypocalcemia with ≤ 2.19 mmol/L [10]; and hypoglycemia with glucose ≤ 3.3 mmol/L [7]. Age and sex specific creatinine reference ranges were used to classify acute kidney injury according to the Kidney Disease Improving Global Outcomes recommendations [15].

### Statistics

Categorical variables are presented as counts and were evaluated using the Fisher exact test. The D’Agostino-Pearson omnibus test for normality revealed that age, O_2_-saturation, body temperature, heart rate, blood pressure, and laboratory parameters did not conform to a Gaussian distribution [16]. Continuous data are therefore shown as median and interquartile range. For analysis, the non-parametric Mann–Whitney-Wilcoxon U test and the Kruskal–Wallis H test, followed by Dunn's post-hoc multiple comparison, were applied. A two-sided *P*-value below 0.05 was deemed statistically significant.

## Results

### Included and excluded cases

Between 2013 and 2023, the diagnosis of binge drinking was established in 305 individuals, 11.3 to 18.4, median 16.4 years of age (female-to-male ratio 0.92). A total of 173 (57%) individuals who met the inclusion criteria were analyzed. Included and excluded patients showed no significant differences in age (16.4 [15.6–17.1] versus 16.4 [15.1–17.1] years; *P* = 0.2459), female-to-male ratio (0.94 versus 1.31; *P* = 0.1661), and O_2_-saturation (98 [97–100] versus 99 [96–100] %; *P* = 0.1254).

### Findings in included cases

#### Level of consciousness

Patients with a normal (*N* = 110) or a reduced Glasgow Coma Scale (*N* = 63) did not significantly differ for demographics, body temperature and O_2_-saturation. Heart rate (*P* = 0.007) and blood pressure (*P* = 0.0091) were slightly higher in cases with normal than those with diminished alertness (Table 1).

**Table 1:** Clinical and blood parameters in 173 subjects 13.2 to 18.0 years of age with an acute episode of episodic heavy ethanol use based on level of consciousness. Results are presented either as value (and percentage) or as median [and interquartile range]. Parameters with statistically different results between groups are presented in bold

			G l a s g o w C o m a S c a l e		
		All cases	≤ 12	13–15	*P*-value
N		173	110	63	
Female-to-male ratio		0.94	1.04	0.80	0.4334
Age, years		16.4 [15.6–17.1]	16.4 [15.6–17.3]	16.2 [15.5–16.8]	0.0964
History of binge drinking		20 (12)	14 (13)	6 (9.5)	0.4510
Substances other than alcohol		23 (13)	18^*^ (16)	5^**◇**^(7.9)	0.1624
Seizures		0 (0)	0 (0)	0 (0)	> 0.999
Body temperature					
Value, °C		36.0 [36.0–36.3]	36.0 [36.0–36.4]	36.0 [36.0–36.2]	0.1005
≤ 35.0		2 (1.2)	2 (1.8)	0 (0)	0.5342
O_2_-saturation					
Value, %		98 [97–100]	99 [98–100]	98 [97–99]	0.1802
Hypoxemia (≤ 92%)		1 (0.6)	0 (0)	1 (1.6)	0.3642
**Heart rate, /min**		**85 [75–98]**	**89 [78–100]**	**80 [71–90]**	**0.0007**
**Mean blood pressure, mm Hg**		**81 [71–88]**	**83 [73–90]**	**78 [70–85]**	**0.0091**
**Ethanol level, g/L**		**1.92 [1.56–2.30]**	**1.72 [1.39–2.16]**	**2.17 [1.91–2.50]**	** < 0.0001**
Albumin, g/L		48 [46–50]	48 [46–50]	48 [46–50]	0.8105
Hemoglobin, g/L		136 [126–146]	135 [126–146]	137 [129–146]	0.2450
Ionized sodium					
Value, mmol/L		140 [138–142]	140 [138–142]	140 [138–142]	0.7413
≤ 134 mmol/L		7 (4.0)	2 (1.8)	5 (7.9)	0.1007
≥ 146 mmol/L		3 (1.7)	2 (1.8)	1 (1.6)	> 0.999
Ionized chloride					
Value, mmol/L		104 [102–106]	104 [102–106]	103 [102–107]	0.6988
≤ 97 mmol/L		0 (0)	0 (0)	0 (0)	> 0.999
≥ 110 mmol/L		0 (0)	0 (0)	0 (0)	> 0.999
Ionized potassium					
Value, mmol/L		3.6 [3.4–4.0]	3.7 [3.4–4.1]	3.5 [3.2–3.9]	0.1455
** ≤ 3.4 mmol/L**		**57 (33)**	**29 (26)**	**28 (44)**	**0.0187**
≥ 5.1 mmol/L		0 (0)	0 (0)	0 (0)	> 0.999
Total calcium					
Value, mmol/L		2.35 [2.28–2.40]	2.36 [2.28–2.40]	2.33 [2.27–2.38]	0.2450
≤ 2.19 mmol/L		0	0	0	> 0.999
≥ 2.61 mmol/L		0	0	0	> 0.999
Glucose					
Value, mmol/L		6.0 [5.4–6.6]	6.0 [5.4–6.5]	6.0 [5.5–6.6]	0.6205
≤ 3.3 mmol/L		0	1	0	> 0.999
Osmolarity					
** Total, mosm/L**		**334 [325–341]**	**329 [320–337]**	**337 [332–345]**	** < 0.0001**
Effective, mosm/L		286 [283–290]	286 [283–289]	286 [282–290]	0.6590
Urea, mmol/L		4.3 [3.3–5.2]	4.0 [3.3–5.0]	4.5 [3.6–5.3]	0.0795
Creatinine					
Value, µmol/L		65 [59–74]	65 [57–74]	68 [61–78]	0.1251
Acute kidney injury		0	0	0	> 0.999

*cannabinoids (*N* = 17), cocaine (*N* = 1); ^**◇**^cannabinoids in all 5 cases

The ethanol level and the total osmolarity were higher (*P* < 0.0001) in patients with an impaired state of awareness (Table 1). Hypokalemia was noted in one third of the cases and was more frequent (44% versus 26%; *P* = 0.0187) among individuals with normal level of consciousness. Albumin, hemoglobin, sodium, chloride, total calcium, glucose, effective osmolarity, urea and creatinine were similar in the two groups. Hypoglycemia occurred in only one case.

#### Acid–base balance

**Fig. 1:**
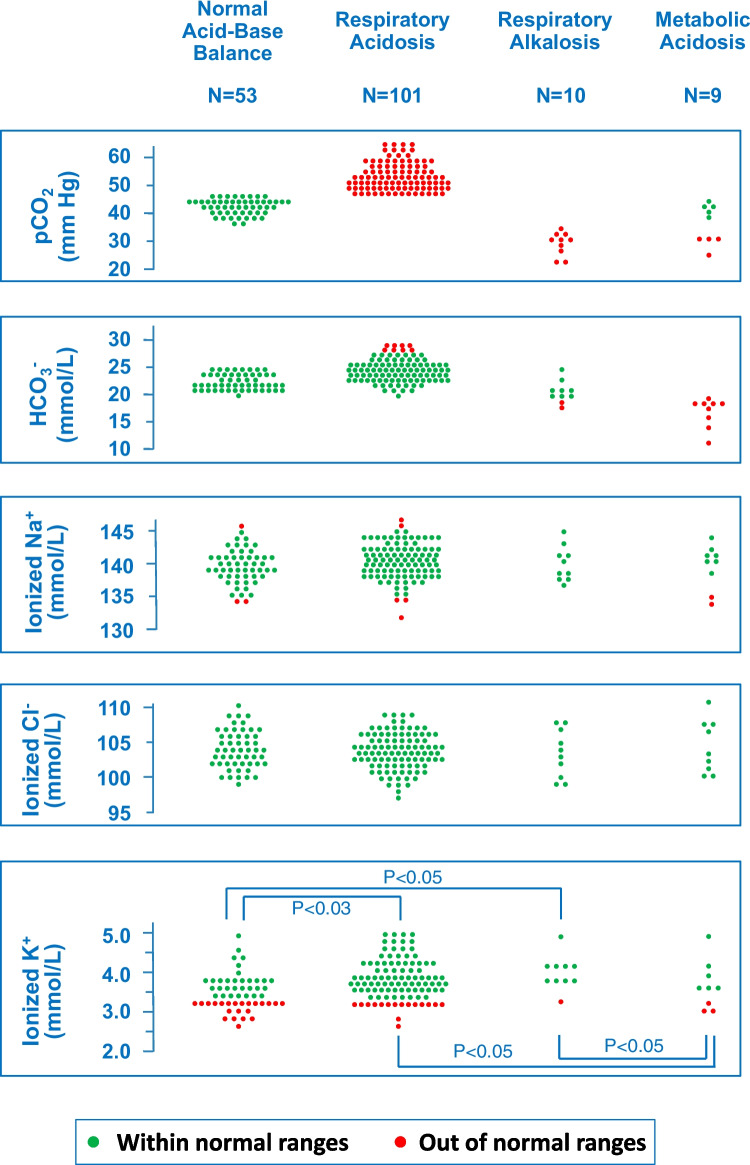
Carbon dioxide pressure, bicarbonate, ionized sodium, ionized chloride and ionized potassium in 173 adolescents with an episode of binge drinking based on acid–base balance. The four groups were not compared with respect to carbon dioxide pressure and bicarbonate, because a statistical difference was anticipated. Sodium and chloride levels showed no significant differences among the four groups. Potassium levels were lower in normal acid–base balance than in respiratory acidosis (*P* < 0.05) and respiratory alkalosis (*P* < 0.05). Similarly, potassium levels were lower in metabolic acidosis than in respiratory acidosis (*P* < 0.05) and respiratory alkalosis (*P* < 0.05)

The acid–base balance appeared normal in 53 (31%) and altered in the remaining 120 (69%) cases (Table 2 and Fig. 1). Specifically, acute respiratory acidosis was observed in 101 (58%), respiratory alkalosis in 10 (5.8%), and metabolic acidosis in 9 (5.2%) cases. Blood anion gap was higher (*P* = 0.0021) in the latter group compared to the remaining group. In patients with metabolic acidosis, lactate level was elevated, ranging from 2.2 to 6.1, with a median of 3.9 mmol/L, indicating the diagnosis of lactic acidosis. The total osmolarity was higher (*P* = 0.0213) in patients with respiratory acidosis compared to the remaining groups.

**Table 2:** Clinical and blood parameters in 173 subjects 13.2 to 18.0 years of age with an episode of heavy ethanol use based on acid–base balance. Results are presented either as value (and percentage) or as median [and interquartile range]. The groups were not compared in terms of acid–base balance because a statistically significant difference is anticipated because of the clinical definition of these disorders. Parameters with statistically different results between groups are presented in bold

		Acid–Base Balance Normal	Respiratory Acidosis	Respiratory Alkalosis	Metabolic Acidosis	P-Value
N		53	101	10	9	
**Female-to-male-ratio**		**1.94**	**0.60**	**1.00**	**2.00**	**0.0059**^✢^
Age, years		16.4 [15.4–17.0]	16.3 [15.5–17.2]	16.8 [16.5–17.8]	16.2 [15.8–16.8]	0.1879
Glasgow coma scale						
Value		**14 [12–15]**	**13 [10–15]**	**15 [14–15]**	**12 [12–15]**	< 0.0001*
≤ 12		17 (32)	40 (40)	1 (10)	5 (56)	0.1319
O_2_-saturation						
Value, %		98 [97–100]	98 [97–99]	100 [99–100]	98 [98–99]	0.1206
Hypoxemia (≤ 92%)		0 (0)	1 (0.99)	0 (0)	0 (0)	> 0.999
Heart rate, /min		92 [77–100]	83 [75–92]	89 [81–104]	86 [75–95]	0.0935
Mean blood pressure, mm Hg		82 [71–88]	79 [70–87]	84 [80–91]	80 [75–87]	0.1945
Ethanol level, g/L		1.88 [1.37–2.26]	2.01 [1.66–2.30]	1.57 [1.42–1.79]	1.97 [1.60–2.59]	0.0566
pH		7.36 [7.33–7.38]	7.30 [7.28–7.32]	7.49 [7.45–7.53]	7.30 [7.28–7.33]	
pCO_2_, mmHg^§^		42 [40–44]	51 [48–56]	30 [26–31]	38 [30–42]	
Bicarbonate, mmol/L		22 [21–24]	25 [23–26]	21 [20–21]	19 [17–19]	
**Hemoglobin, g/L**		**130 [123–141]**	**134 [130–147]**	**142 [133–149]**	**133 [131–138]**	**0.0110**^✢^
**Albumin, g/L**		**47 [45–49]**	**48 [46–50]**	**50 [49–52]**	**47 [45–49]**	**0.0078**^**✢**^
Ionized sodium, mmol/L		139 [138–141]	140 [139–142]	139 [137–141]	140 [138–141]	0.3766
Ionized chloride, mmol/L		104 [102–106]	104 [102–106]	104 [101–107]	104 [102–108]	0.8688
**Ionized potassium**						
** value, mmol/L**		**3.4 [3.1–3.7]**	**3.8 [3.5–4.2]**	**3.7 [3.5–3.8]**	**3.5 [3.1–4.1]**	**0.0049**^**†**^
** ≤ 3.4 mmol/L**		**29 (54)**	**24 (24)**	**1 (10)**	**3 (33)**	**0.0006**^**†**^
**Total calcium, mmol/L**		**2.35 [2.27–2.39]**	**2.34 [2.27–2.40]**	**2.48 [2.35–2.56]**	**2.36 [2.28–2.38]**	**0.0492**^✢^
**Anion gap, mmol/L**		**17 [15–19]**	**15 [13–17]**	**19 [17–21]**	**21 [17–25]**	**0.0021**^▵^
Osmolarity, mosm/L						
** Total**		**330 [320–338]**	**337 [326–341]**	**327 [319–331]**	**332 [331–343]**	**0.0213**^**‡**^
Effective		285 [281–288]	287 [283–290]	286 [283–288]	285 [283–288]	0.1844

^**✢**^ normal balance and metabolic acidosis versus respiratory acidosis and respiratory alkalosis; * normal balance and respiratory alkalosis versus respiratory acidosis and metabolic acidosis; ^✢^ respiratory alkalosis versus the remaining groups; ^†^ normal balance and metabolic acidosis versus respiratory acidosis and respiratory alkalosis; ▵ metabolic acidosis versus remaining groups; ^‡^ respiratory acidosis versus normal balance and respiratory alkalosis; ^§^ to convert pCO₂ from mmHg kPa, multiply by 0.133

Patients with normal and altered acid–base balance did not show significant differences in age, heart rate, O_2_-saturation, blood pressure, ethanol level, ionized sodium, ionized chloride, and glucose. Females exhibited a higher prevalence (*P* = 0.0059) of both normal acid–base balance and metabolic acidosis.

The Glasgow coma scale was significantly (*P* < 0.0001) lower in the two groups of patients with an acute acidosis (both respiratory and metabolic) as compared with those with a normal acid–base balance and respiratory alkalosis. Hemoglobin (*P* = 0.0110), albumin (*P* = 0.0078) and total calcium (*P* = 0.0492) were significantly higher in patients with acute respiratory alkalosis as compared with the remaining three groups.

Potassium level was on the average lower (*P* = 0.0004) and the prevalence of hypokalemia higher (*P* = 0.0004) in normal acid–base equilibrium and metabolic acidosis compared to respiratory acidosis and respiratory alkalosis. The bicarbonate level was similar (*P* = 0.0768) in individuals with (23 [22–25] mmol/L) and without hypokalemia (24 [22–26] mmol/L).

Respiratory support beyond O_2_-therapy was never needed.

## Discussion

This study encompasses an analysis of over 170 adolescents who engaged in binge drinking [2] and exhibited a median blood ethanol concentration marginally below 2.0 g/L. To summarize: 1. a diminished level of consciousness was observed in 36% of cases; 2. hypothermia and hypoxemia were rare; 3. hypoglycemia and altered sodium and calcium levels were rarely (or never) noted; 4. acute respiratory acidosis was prevalent (58%), while respiratory alkalosis (5.8%) and metabolic acidosis (5.2%) were sometimes noted (no cases of metabolic alkalosis were identified); 5. hypokalemia occurred in one-third of the cases and was less pronounced in respiratory acid–base disturbances.

The discussion will focus on hypoglycemia and dysnatremia, the acid–base disturbances and the prevalence of hypokalemia.

Alcohol consumption is considered a relevant factor in causing hypoglycemia among non-diabetics [6, 7, 9, 17]. This laboratory abnormality particularly arises in infants and young children following acute excessive ethanol consumption [1, 3, 18, 19], as well as in adults with chronic alcohol-use disorder [6, 7, 9, 17]. The present analysis demonstrates that hypoglycemia is rare in previously healthy adolescents after binge drinking [20]. Nonetheless, glucose monitoring is advised, especially in cases with an altered mental status [12]. In malnourished individuals with chronic alcohol-use disorder, consuming substantial amounts of fluids, primarily beer, can impair electrolyte-free water excretion, leading to severe dilutional hyponatremia and low effective blood osmolarity [8, 9, 21]. The considered case series showed no instances of this disturbance, known as beer potomania, likely due to the adequate nutritional status of the included cases.

Acute respiratory acidosis was the most common acid–base abnormality in this case series [8]. This is likely attributed to adolescents often consuming high amounts of alcohol per occasion, reflected in an ethanol concentration of around 2 g/L [2]. Acute respiratory alkalosis, which occurs rather often during withdrawal after chronic alcohol use disorder, was observed in a minority of cases [8]. Anion gap metabolic acidosis (or “Acute anion gap metabolic acidosis”), at least partially due to lactate accumulation, was also observed, as previously reported in the literature on binge drinking [9, 20, 22, 23]. The latter association is less known than that between long-standing alcohol dependence and ketoacidosis [6, 9, 17, 23]. Binge drinking is suggested to cause metabolic alkalosis through vomiting, but no such cases were identified in the included records [2]. Subjects experiencing acute respiratory alkalosis showed elevated hemoglobin, albumin, and total calcium levels, likely due to hyperventilation causing a reduced intravascular volume [24, 25].

**Fig. 2:**
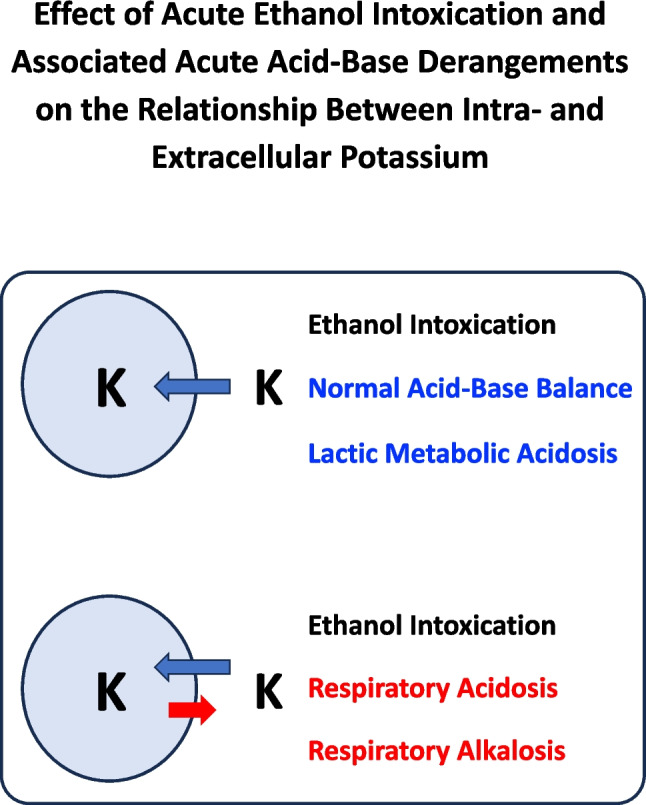
Effects of acute ethanol intoxication and associated acute acid–base derangements on intra- and extracellular potassium level. The two blue circles denote the cell. The blue arrows denote a potassium flow from the extra- to the intra-cellar compartment, the red arrow a flow from the intra- to the extra-cellular compartment (observed only in cases with respiratory acidosis and alkalosis)

Hypokalemia occurred in every third individual with acute binge drinking, as previously described both in adults [5, 9, 26, 27] and in adolescents [20, 28–32]. A reduced potassium intake is unlikely as an explanation, as the participants were previously healthy [33]. Vomiting, commonly associated with binge drinking [2], can cause hypokalemia. However, since vomiting-induced hypokalemia usually accompanies metabolic alkalosis and no cases of this acid–base imbalance were observed, we suggest that vomiting was not a major cause in our series. Given that acute administration of ethanol to normal subjects is followed by decreased urinary potassium excretion [26, 27], it is speculated that hypokalemia results from an increased entry into cells (Fig. 2, upper panel).

Hypokalemia was significantly less common in cases with acute respiratory acidosis [34] and acute respiratory alkalosis [25, 35, 36]. These acid–base disorders result in an increment in blood potassium concentration due to a leakage of this ion from the intra- to the extracellular compartment (Fig. 2, lower panel). Of note, it is often erroneously assumed that acute respiratory alkalosis, like metabolic alkalosis, lowers potassium levels [36]. Although we have not observed cases of binge drinking associated with metabolic alkalosis, we speculate that the tendency for hypokalemia should be particularly relevant in this instance. Hypokalemia prevalence was similar in patients with lactic acidosis and those with normal acid–base balance, implying no potassium leakage from intra- to extracellular compartments in acute metabolic acidosis secondary (Fig. 2, upper panel) to an elevated acid production [34, 35].

This study has several limitations. First, its retrospective design may have introduced selection bias, as only cases with complete data were included. Second, the data were collected from a single center, which may limit the generalizability of the findings to other populations or settings. Third, no systematic screening for the use of illicit substances was conducted, which may have influenced the results. Finally, the study did not explore the long-term outcomes of the biochemical disturbances observed, nor did it assess potential recurrence of binge drinking episodes in these individuals. This pre-registered study also has notable strengths. It includes a large cohort of adolescents with acute ethanol intoxication, offering a detailed analysis of their clinical and biochemical profiles. By focusing on healthy adolescents, it eliminates confounding factors from pre-existing conditions. The study provides valuable insights into glucose, electrolyte, and acid–base homeostasis, and standardized testing methods ensure reliable results [37, 38].

This work investigated adolescents who engaged in binge drinking, focusing on sodium, potassium, calcium, and acid–base balance, which were measured in blood. Future research could benefit from including measurements of magnesium and phosphate, assessing the urinary electrolyte excretion, and analyzing not only total but also ionized calcium and magnesium in blood [39, 40].

## Conclusions

In this cohort of adolescents who engaged in binge drinking, a disrupted acid–base balance and hypokalemia were common. In contrast, glucose, sodium and calcium levels were rarely affected.

## Data Availability

Data ara available upon resonable request to the corresponding author.

## Declarations

### Data Access Statement

The data supporting the findings of this study can be obtained from the corresponding authors upon appropriate request.

### Generative Artificial Intelligence

Generative artificial intelligence was used to improve readability and language of the manuscript.
